# Nutritional value of raw soybeans, extruded soybeans, roasted soybeans and tallow as fat sources in early lactating dairy cows

**Published:** 2012-09-26

**Authors:** H. Amanlou, N. Maheri-Sis, S. Bassiri, A. Mirza-Aghazadeh, R. Salamatdust, A. Moosavi, V. Karimi

**Affiliations:** 1 *Department of Animal Science, Faculty of Agriculture, Zanjan University, P.O.Box 45195-313, Zanjan, Iran*; 2 *Department of Animal Science, Shabestar Branch, Islamic Azad University, Shabestar, East Azerbaijan, Iran*; 3 *Department of Animal Science, Faculty of Agriculture, Urmia University, Urmia, Iran*; 4 *Azar Negin Coorporation, East Azerbaijan, Azarshahr, Iran*

**Keywords:** Dairy cows, Fat supplements, Oilseeds, Whole soybean

## Abstract

Thirty multiparous Holstein cows (29.8 ± 4.01days in milk; 671.6 ± 31.47 kg of body weight) were used in a completely randomized design to compare nutritional value of four fat sources including tallow, raw soybeans, extruded soybeans and roasted soybeans for 8 weeks. Experimental diets were a control containing 27.4 % alfalfa silage, 22.5% corn silage, and 50.1% concentrate, and four diets with either tallow, raw soybean, extruded soybean, or roasted soybean added to provide 1.93% supplemental fat. Dry matter and NEL intakes were similar among treatments, while cows fed fat diets had significantly (*P*<0.05) high NEL intakes when compared to control with no fat. Supplemental fat, whether tallow or full fat soybeans increased milk production (1.89-2.45 kg/d; *P*<0.01) and FCM production (1.05-2.79; *P*<0.01). Milk fat yield and percentage of cows fed fat-supplemented diets were significantly (*P*<0.01 and *P*<0.05 respectively) higher than control. Between fat-supplemented diets, roasted soybean caused highest milk fat yield and extruded soybean caused lowest milk fat yield. There was no significant effect of supplemental fat on the milk protein and lactose content and yield. Feed efficiency of fat-supplemented diets was significantly (*P*<0.01) higher than control. Body weight, body weight change and BCS (body condition score) of cows, as well as energy balance and energy efficiency were similar between treatments. In conclusion, while there was no significant effect of fat sources on production response of cows, fat originating from heat-treated soybean help to minimize imported RUP (rumen undegradable protein) sources level as fish meal in comparison with tallow and raw soybean oil. In the Current study, there was no statistical significance among nutritional values of oil from extruded soybeans and roasted soybeans.

## Introduction

In recent decades, there has been an increasing interest in feeding dairy cows oilseeds, because besides protein, oilseeds are valuable sources of energy in the form of fat. Fat can increase the energy density of dairy diets without the ruminal acidosis and depressed milk fat tests of high starch, low-fiber diets (Coppock and Wilks, 1991). Moreover, the high energy density of supplemental fats allows for greater energy consumption and direct transfer of fatty acids of the supplemental fats to milk fat; this increases metabolic efficiency (Coppock and Wilks, 1991). Encapsulation of fatty acids within the oilseed has additional benefit of lessening detrimental effect of fat on digestion, which is the major limiting factor in fat utilization (Jenkins and Lundy, 2001). Full fat soybeans are one of the major oilseeds consisting of 17 to 20% oil on a dry matter basis. *Soybeans* differ from the *other oilseeds* such as canola, sunflower, and safflower seeds, as those seeds contain more oil that is more highly unsaturated and, therefore, lower amounts should be fed to cows, for example, only half as much canola should be fed as compared to soybeans (Amaral-Phillips *et al.*, 1997). Moreover, soybeans are a very palatable and are easily fed in different feeding systems, such as top dressed on silages, part of the total mixed ration, or in a grain mix (Amaral-Phillips *et al.*, 1997). Soybeans may be successfully fed to dairy cows raw, roasted or extruded; and whole, rolled or ground. Grinding or rolling may increase utilization, depending on the total ration, and may make handling easier. However, grinding or rolling may increase risk of rancidity for raw soybeans. The type of processing may affect oil release in the rumen and influence utilization (Reddy *et al.*, 1994).

Extrusion has been shown to increase the ruminal availability of soy oil; in contrast, roasting may slow the rate of release of soy oil into the rumen (Scott *et al.*, 1991(. Privé *et al*. (2010) proposed processing oilseeds with heat may have effects on ruminal lipid digestion could be caused by a modification of the seed coat protection, a reduction of the amount of polyunsaturated fatty acids subjected to biohydrogenation, or the production of oxidation products. There are many investigations examining the effect of heat treated soybeans on performance of dairy cows (Faldet and Satter, 1991; Scott *et al.*, 1991; Chouinard *et al.*, 1997a, 1997b), however, they were primarily designed to examine the effect of heating process on protein degradability. The objective of the current study was to compare nutritional value of oil in raw soybeans, extruded soybean and roasted soybean with tallow (a commonly used fat), by observing the lactation response of dairy cows. In contrast with previous experiments, we tried to supply the same level of RUP in treatments, to avoid the confounding effect of differences in degradability of protein on performance. The 10% soybean level, selected in this experiment, was based on the current feeding guidelines that recommended 10% ration of Organic Matter the full fat soybeans (Scott *et al.*, 1991).

## Materials and Methods

The experiment was conducted at the Azar Negin Coorporation throughout May and June 2011. Thirty multiparous high-producing Holstein cows were selected from the Azar Negin dairy herd based on DIM (days in milk). The selected cows were housed in a tie-stall system, and fed the standard herd ration for the first five days of experiment (pretrial period) for covariate data collection, and were then adapted to the experimental diets for 14 days. At the onset of an eight-week experimental period the cows (DIM: 29.80 ± 4.01) were randomly assigned to one of five treatments being a control diet or a fat-containing diet that included tallow or raw soybeans, or extruded soybeans or roasted soybeans. Afterwards, five diets were formulated to be isocaloric (except the control diet), isonitrogenous and iso-RUP (Table 1). The experimental diets contained about 27.4 % alfalfa silage, 22 % corn silage, and 50 % concentrate, and included no fat (control) or 1.93 % fat originated from tallow, raw soybeans, extruded soybeans, or roasted soybeans replaced some of the corn/soybean meal.

**Table 1:** Ingredient and nutrient composition of experimental diets.

Item, % of DM	Control	1	2	3	4
Ingredient					
Alfalfa hay	27.38	27.38	27.37	27.42	27.45
Corn silage	22.56	22.56	22.55	22.01	22.04
Corn, ground	24.29	22.36	22.36	23.56	23.58
Tallow	-	1.93	-	-	-
Corn gluten feed	6.94	6.94	6.94	8.11	8.12
Soybean meal	8.10	8.10	-	-	-
Raw soybean	-	-	10.02	-	-
Roasted soybean	-	-	-	-	10.05
Extruded soybean	-	-	-	10.04	-
Fishmeal	4.78	4.78	4.96	1.86	1.74
Meat meal	0.77	0.77	0.77	0.77	0.77
Calcium PhosDi	0.39	0.39	0.39	0.39	0.39
Molasses	3.86	3.86	3.86	2.70	2.70
Fish oil	-	-	-	0.39	0.39
Urea	.15	.15	-	0.62	0.64
Mineral-vitamin mix	0.78	0.78	0.78	0.78	0.78
Wheat straw	-	-	-	1.35	1.35
Nutrient					
DM, %	55.4	56.33	56.27	57.04	57.00
CP, % of DM	18.6	18.5	18.5	18.5	18.5
RDP*, % of CP	58.9	59	58.7	58.4	58.4
RUP*, % of CP	41.1	41	41.3	41.6	41.6
NDF, % of DM	28.9	28.8	28.9	29.6	29.6
NFC^1^, % of DM	44.3	42.8	42.8	43.2	43.3
Ether extract, % of DM	3.5	5.4	5.1	5.3	5.3
Calcium, % of DM	0.9	0.96	0.9	0.73	0.72
Phosphorus.% of DM	0.61	0.55	0.63	0.54	0.52
NE_L_*, Mcal/kg DM	1.69	1.75	1.74	1.72	1.72

1, 2, 3, 4 are respectively: diet containing 1.93% tallow, 10% raw soybeans, 10% extruded soybeans and 10% roasted soybeans.

* Calculated using the CPM-DairyV3computer program from composition of feedstuffs.

1 NFC = 100 − (NDF + CP + ether extract + ash).

The whole raw and roasted soybeans used in the trial were purchased from Tehran Daneh Cooperation (Tehran, Iran). Roasted whole soybeans were prepared by heating soybeans to 145°C in an automatic Roaster Jet. Apparatus, with a cooling time of 3 h. Extruding process consisted of grinding, conditioning, extruding, cooling and storage. Extrusion of soybean was conducted at 155-160°C for 15-20 s. The ground extruded soybeans were obtained from Salehkashmar Cooperation (Mashhad, Iran). Diets were fed daily at 0700 and 0900, 1100, 1500 and 2100 h as a total mixed ration for ad libitum intake throughout the experiment (target of 10% orts). Tallow was first heated and then mixed thoroughly with the concentrate portion of the diet before feeding. Feed offered and orts were weighed daily for individual cows in the pretrial period as well as the experimental period. Samples of TMR and orts were collected once in the pretrial period and weekly during the experimental period and dried at 105°C for 24 h for DM determination. Daily DMI was determined based on the DM concentration of the TMR offered and orts. Dry matter content of corn silage and feed ingredients was determined weekly and diets were adjusted to maintain proportions of each on a DM basis. Monthly composite samples of individual feed ingredients were analyzed for CP, EE and ash contents by standard methods (AOAC, 1990). Analyses of NDF and ADF were performed according to the methods of Van Soest *et al.*, 1991. Chemical composition of the diets was calculated from chemical analyses of the individual ingredients.

Cows were milked three times at 0600, 1400 and 2200 h daily, and individual weights were recorded at each milking throughout the pretrial and experimental periods. A composite milk sample was prepared from each cow from the three consecutive milkings, twice in the pretrial period and weekly in the experimental period. Samples were sent to the Research Station of Indigenous Sarabi cattle herd (Sarab-Iran) for fat, protein and lactose content determination (Milk analyzer Dairyscan Jet1, Bulgaria).

Blood samples (10 ml) were taken by venoject from the jugular vein once every *two weeks* at 1500 h. Samples were allowed to clot (1 to 2 h) at room temperature (25°C) and then centrifuged for 30 min at 1700 *g* at 4°C. Serum was collected and sent to a commercial laboratory (Laboratory for Veterinary Medicine, Karaj, Iran) for NEFA (non-esterified fatty acids) and BHBA (β -hydroxybutyric acid) measurement. Serum concentrations of NEFA and BHBA were measured using *kits* from *Randox* (Crumlin, UK) by enzymatic methods.

Cows were weighed and body condition scored every 2 weeks. Weighting was conducted on two day consecutively before feeding and the mean calculated. Body condition scores were determined on a Scale of 1 to 5 (Wildman *et al.*, 1982).

Pretrial values for DMI, milk yield, BW and BCS were reduced to means and used as covariates during statistical analy. Data were analyzed using the PROC MIXED procedure of SAS (version 9.2, SAS Institute Inc., Cary, NC, US) for a completely randomized design with repeated measures with the following model:

Yijk = µ + *α*i + *β*j + *δ*l + *βδ*jl + C + *ε*ijl

where Yijk is dependent variable, µ is overall mean, *α*i is random effect of the cow, *β*j is the fixed effect of the treatment, *δ*l is the fixed effect of the measurement, *βδ*jl is the fixed effect of interaction between *β*j and *δ*l, C is the fixed effect of appropriate covariate (e.g., milk yield, milk composition, or BW) and *ε*ijl is the residual error. Tukey’s test was used to test treatment means (*P* < 0.05) of the experiment.

## Results

The ingredient and chemical composition of the experimental diets are presented in Table 1. The diets were formulated to contain approximately 18.5% CP, 29% NDF, and 41% RUP (Table 1). The effects of treatment and fat supplements on lactation performance of dairy cows are shown in Table 2. The covariate effect was detected for some parameters, however because the various covariates were used for different parameters, the values was not shown in a table.

**Table 2:** Effects of different fat sources on lactation performance and efficiency of dairy cows.

Item	Treatments					SEM	*P*-value			
	Control	1	2	3	4		Treatment	Fat vs Control	Week	Treatment*week
DMI, kg/d	24.27	23.91	24.29	24.41	24.91	0.25	0.1916	0.7148	<.0001	0.5434
Milk yield, Kg/d	43.66	45.55	45.55	45.77	46.11	0.57	0.0850	0.0087	0.0005	0.0337
3.5% FCM^1^	41.46b	43.57a	43.87a	43.28a	44.25a	0.48	0.0208	0.0022	0.0006	0.0442
FCM/DMI	1.70b	1.82a	1.79a	1.77 a	1.78a	0.019	0.0217	0.0024	<.0001	0.0258
Milk fat, %	3.10	3.23	3.30	3.17	3.31	0.05	0.1089	0.0304	0.1429	0.3316
Milk fat, Kg/d	1.35b	1.46a	1.49a	1.45a	1.51a	0.022	0.0039	0.0004	0.0038	0.5276
Milk protein, %	3.04	3.03	3.06	2.86	2.83	0.08	0.3364	0.3584	0.4013	0.5576
Milk protein, Kg/d	1.32	1.38	1.39	1.31	1.30	0.04	0.5728	0.6889	0.0691	0.0612
Milk lactose, %	5.07	4.73	5.05	4.88	4.70	0.13	0.2324	0.1485	0.0074	0.0010
Milk lactose, Kg/d	2.20	2.14	2.30	2.23	2.17	0.004	0.3150	0.3824	0.0894	0.0021
Body weight, kg	657.62	652.54	656.70	655.86	661.21	6.1	0.9008	0.8293	<.0001	0.9824
BW change, kg^2^	-4.09	-4.20	-4.24	-4.76	-4.54	1.05	0.9897	0.7768	<.0001	0.6855
Energybalance^3^, Mcal/day	+2.27	+1.74	+1.84	+1.96	+1.99	0.47	0.9178	0.4318	<.0001	0.2236
NE_L_ intake, Mcal/day	41.06	41.90	42.28	41.98	42.82	0.41	0.1168	0.0281	<.0001	0.5196
Energy efficiency^4^	0.96	0.97	0.96	0.95	0.97	0.007	0.1787	0.1699	<.0001	0.0024
BCS	2.52	2.51	2.48	2.50	2.46	0.02	0.6106	0.7641	<.0001	0.2205
BHBA	0.484	0.430	0.378	0.383	0.368	0.045	0.0935	0.0152	<.0001	0.1443
NEFA	0.254	0.245	0.239	0.241	0.228	0.01	0.5816	0.1888	0.0590	0.6159

1, 2, 3, 4 are respectively: diet containing 1.93% tallow, 10% raw soybeans, 10% extruded soybeans and 10% roasted soybeans.

1 3.5% FCM = 0.432 (kilograms of milk) + 16.2 (kilograms of fat),

2 two weekly means,

3 Energy balance = NE_L_ intake - NE_L_ for maintenance and milk yield,

4 Energy efficiency = NE_L_ output/NE_L_ intake, NE_L_ Output = NE_L_ for maintenance, milk yield, and BW change.

DMI of cows was not significantly affected by treatments or fat sources, but cows fed roasted soybean diets had numerically higher DMI, and cows fed tallow diets had numerically lower intakes than cows fed other diets. There was no effect of treatment on NE_L_ intakes, while cows fed the fat diets had higher NE_L_ intakes (*P*<0.05) compared to cows fed the control diet. Supplemental fat, whether tallow or full fat soybeans, increased milk yield (1.89-2.45 kg/d; *P*<0.01) and FCM production (1.05-2.79 kg/d; *P*<0.01). Cows fed roasted soybeans had numerically higher FCM than for cows fed other fat diets. Feed efficiency of the fat supplemented diets (1.82, 1.79, 1.77 and 1.78 for tallow, raw soybean, extruded soybean and roasted soybean, respectively) were significantly (*P*<0.01) higher than the control (1.70), however, there were no significant differences between fat-supplemented diets. Milk fat percentages (*P*<0.05) and yield (*P*<0.01) were higher for cows fed the fat diets than cows fed the control diet with no fat. Comparing the fat diets, cows fed roasted soybeans had numerically higher percentages and yield of milk fat than cows fed other fat diets (Table 2). Milk protein percentages and yield were not affected by treatment or fat supplementation. Body weight change of cows and energy balance were similar for cows fed all diets (Table 2). Serum NEFA and BHBA concentrations were similar among treatments; however cows fed fat diets had significantly (*P*<0.05) lower BHBA concentrations when compared to control with no fat (Table 2).

## Discussion

The lack of fat effect due to heated soybean (Bernard, 1990; AbuGhazaleh *et al.*, 2004; Rong *et al.*, 2010) or tallow (Grummer *et al.*, 1993; Miller *et al.*, 2009) on DMI has been previously reported. Supplemental fat potentially limits feed intake by reducing fiber digestion and passage from the rumen (Grummer *et al.*, 1993); however, the effects of level and type of fat supplement on DMI are negligible when total dietary fat concentration is below 6% of the dry matter (Petit, 2010).

Moreover, whole oilseeds lessen the severity of digestion problems by encapsulation of anti-microbial fatty acids within their hard outer seed coat (Jenkins and Lundy, 2001).

Increased NE_L_ intake for cows fed the fat diets compared to cows fed the control diet may be due to slight differences in NE_L_ content of diets or DMI of cows (Tables 1 and 2). The higher feed efficiency for fat diets may be attributed to the increasing nutrient digestibility of fat diets. Increased nutrient digestibility for rations containing supplemental fat was reported by previous researchers (Palmquist and Comad, 1978; Bernard, 1990; Gonthier *et al.*, 2004).

Weiss *et al*. (2011) stated fat-supplemented diets had more DE (2.93 Mcal/kg) than the control diet (2.83 Mcal/kg), and DE intake by cows fed supplemented diets was 1.6 Mcal/d greater than by cows fed the control diet. Higher fiber and lower starch concentrations of rations containing fat compared with control would favor a more stable rumen environment and fiber digestibility. Moreover, conventional diets contain greater quantities of waxes, sterols, and terpenoids, which are poorly digested and constitute a large portion of endogenous losses compared with fatty acids, which are highly digestible and comprise a higher percentage of EE in fat-supplemented diets (Bernard, 1990).

Supplemental fat may elevate milk yield and milk fat, but different types and sources of fat may have diverse effects on these parameters (Chouinard *et al.*, 1997a). Heat-treated oilseeds may increase milk yield by a combination of increase in NE_L_ intake and protein delivery to the small intestine (Schmidely *et al.*, 2005). Moreover, the direct incorporation of fatty acids into milk cause a decrease in *de novo* synthesis of fatty acids and a greater proportion of available glucose being used for lactose synthesis and consequently an increase in milk production (Hammon *et al.*, 2008). The RUP concentration remained constant among diets in the current experiment, while NE_L_ intake was numerically greater for cows fed the fat diets.

Percentage and yield of milk fat has been observed to increase when cows were fed whole oilseeds or protected fats (Knapp *et al.*, 1991; Kim *et al.*, 1993; Petit and Gagnon, 2009), but not in all studies (AbuGhazaleh *et al.*, 2004, Miller *et al.*, 2009; Petit, 2010). Increased milk fat content when cows fed fat-supplemented diets may be due to increased dietary fatty acids being taken up by the mammary gland for milk fat synthesis (Knapp *et al.*, 1991).

Similary, Duske *et al*. (2009) suspected that higher milk fat percentages are due to triacylglycerols being transported by chylomicrons and utilized by the mammary gland. A numerical low content and yield of milk fat for extruded soybean-fed cows compared with other soybean diets may be related to more rapid release of oil into the rumen, resulting from extrusion rupturing of the fat micelles (Scott *et al.*, 1991). Under certain circumstances, rumen biohydrogenation of linoleice and linolenic acids results in unique fatty acids, such as *trans*-10, *cis*-12 conjugated linolenic acid that are potent inhibitors of *de novo* fatty acids synthesis (Caldari-Torres *et al.*, 2011). However, milk fat yield for cows fed extruded soybeans was still significantly more (*P*<0.01) than cows fed the control diet, implying that the decrease in milk C4 to C16 fatty acid yield was compensated by an increase in C18 fatty acid yield (Glasser *et al.*, 2008). Moreover, yield and percentage of milk fat was higher when soybean oil was fed continuously (as in the current experiment) than when fed in two meals (Chouinard *et al.*, 1997b).

Supplemental fats, including sources other than oilseeds, generally decrease milk protein percentage (Pires *et al.*, 1996), but results from oilseeds vary. Some researchers reported no change in milk protein content with oilseeds utilization (Bernad, 1990; Chilliard *et al.*, 2009; Radivojević *et al.*, 2011), while others (Faldet and Satter, 1991; Pires *et al.*, 1996; Miller *et al.*, 2009) reported a decrease in milk protein percentage, in particular when cows were fed extruded oilseeds.

The decrease in milk protein percentage might have been due to an increased availability of fat in the rumen and a reduction in duodenal flow of microbial proteins (Gonthier *et al.*, 2004; Petit, 2010). The lack of effect of the fat supplement on milk fat in our experiment supports this hypothesis. On the other hand, Chouinard *et al*. (1997a) suggested that the effect of heat treatment, applied to full fat soybean, might originate at the protein level rather than the lipid fraction of the seed.

Garnsworthy *et al*. (2008) reported that body weight change was not influenced by dietary concentrations of starch and fat. Miller *et al*. (2009) reported no effect of fat supplement (whole cottonseed, tallow, or full-fat corn germ) on body weight change. In Chouinard *et al*. (1997a), replacement of ground raw soybeans by heated soybeans (extruded soybeans, micronized soybeans, and roasted soybeans) had no effect on the body weights of cows at weeks 4 and 8 of the experimental period. Fatty acids may alter energy balance through changes in dry matter intake, nutrient digestibility, and milk and tissue synthesis (Harvatine and Allen, 2006).

In the current study, addition of fat did not improve energy balance or prevent body weight loss. Petit (2010) stated that in the early stage of lactation long-chain fatty acids may be preferentially oxidized, whereas in the mid stage of lactation, when cows are in a more positive energy balance, the supplemental long chain fatty acids may be used for body tissue deposition. The positive energy balance in spite of the body weight loss that was observed in this study might reflect overestimation of the energy content of the feeds as previously reported (Hartnel *et al.*, 1991; Son *et al.*, 1996).

Kronfeld (1982) proposed that dietary fat would decrease mobilization of body fat and help to decrease concentrations of NEFA. Others reported that fat supplementation increased the concentrations of NEFA and/or BHBA in blood of dairy cows (Delbecchi *et al.*, 2001; Petit *et al.*, 2001, Petit, 2002; Boken *et al.*, 2005), or had no effect (Grummer *et al.*, 1993; Pires *et al.*, 1996; Pickett *et al.*, 2003; Petit *et al.*, 2004; Harvatine and Allen, 2006; Cerri *et al.*, 2009). However, concentrations of serum BHBA decreased with supplemental fat, especially soybeans when compared to control in current experiment, which may be due to inhibitory effect of linonenic acid containing in soybeans on lipolysis (Mashek *et al.*, 2005).

## Conclusion

In conclusion, the addition of 1.93% oil from extruded or roasted soybeans added to the diet of early lactating dairy cows, beside supporting equal or higher milk and milk composition, help to minimizing the dietary level of imported bypass protein sources such as fish meal, without any adverse effect on circulating NEFA or BHBA concentrations, when compared to raw soybean or tallow.

There were no significant differences between extruded or roasted soybean oil in lactation performance and energy balance of dairy cows. However, cows fed roasted soybean had numerically greater milk and FCM production and fat content and yield than cows fed extruded soybeans.
